# Gender in the disclosure of loneliness on Twitter during COVID-19 lockdowns

**DOI:** 10.3389/fdgth.2023.1297983

**Published:** 2023-12-06

**Authors:** Yelena Mejova, Anya Hommadova Lu

**Affiliations:** ^1^ ISI Foundation, Turin, Italy; ^2^ Communication Studies, Sam Houston State University, Huntsville, TX, United States

**Keywords:** loneliness, COVID-19, lockdowns, computer mediated communication, social media, self-disclosure, gender

## Abstract

**Introduction:**

Loneliness has been shown to affect both mental and physical health, and was a major concern even before the COVID-19 pandemic. During the COVID-19 distancing measures, millions of people took to social media to express their feelings and seek social support.

**Methods:**

In this mixed-methods study, we examine the self-disclosure of loneliness by users identifying as male or female (via self-disclosed naming conventions) on Twitter before and during the COVID-19 “lockdowns.”

**Results:**

We show that in the first two months of COVID-19 restrictions, self-disclosure of loneliness on this platform rose dramatically, and also have changed qualitatively. We find that female accounts tend to post more loneliness self-disclosures compared to male ones, even before COVID. Female disclosures more often center around pregnancy, family, and close relationships, whereas those posted by the male ones are more related to leadership, video gaming and sex. During COVID lockdowns, female accounts turn to online messaging apps and hobbies, and male become increasingly vocal in seeking partners.

**Discussion:**

The insights of this study have important implications for the design of interventions for lessening the burden of loneliness in the current digitized world.

## 1. Introduction

The unprecedented measures taken during the SARS-CoV-2 (or simply COVID-19) pandemic changed the daily lives of millions, perhaps billions, of people around the world. Local governments everywhere, lead by the recommendations of the public health authorities, instituted physical (sometimes also called “social”) distancing as a part of non-pharmaceutical containment and mitigation strategies necessary before the development and deployment of mass vaccinations. Although eventually proven to be on the whole an effective strategy (1), what came to be known as the “lockdowns” have had a profound impact on the economic, social, and personal being of multitudes. It is now known that these measures have resulted in a heightened levels of depression and anxiety (2–4), especially amongst the students and healthcare professionals (5). Although the situation contributed many complex causes for the ensuing mental health challenges, the experience of loneliness is likely to be an important factor (6). According to Masi et al. (7), “The associations between loneliness and physical and mental health indicate that loneliness influences virtually every aspect of life in our social species”(p. 220). A recent study has found that one-in-three Canadians developed severe loneliness between march and may 2020 (8), while at the same time in Hong Kong, an “astonishing 65.6% of the respondents reported clinical levels of depression, anxiety, and/or stress” (9). Another study in the U.S. during the COVID-19 pandemic showed a significant increase in loneliness scores particularly from April to May 2020 (10). Even before the COVID-19 pandemic, loneliness has been called an “epidemic” in the United States (11). It is associated with a slew of physical and mental problems, including high blood pressure, an increased risk of Alzheimer’s disease, as well as suicidal ideation and depression (12–14).

The evolutionary perspective on loneliness suggests that the pain experienced by loneliness serves the purpose of motivating people to form connections with others. Through this theoretical lens, loneliness is viewed as a biological construct, and helps people be aware of potentially dangerous social threats and motivates one to renew connections needed to survive, and increase the likelihood of his or her genes making it into the gene pool (15, 16). There are several explanations for the experience of loneliness and its evolutionary necessity according to various scholars: (1) reproduction (loneliness is negatively associated with having a partner), (2) survival of offspring (parent share resources with children and feel lonely when separated), and (3) preservation/survival through social network (higher chance of survival in groups) (17, 7). In the case of COVID-19 pandemic, with social isolation and lockdown restrictions there were fewer ways to remedy loneliness.

Therapy and self-expression may be one way to alleviate the experiences of loneliness (18). However, self-disclosure of loneliness may be stigmatized, and perceived as self-absorbed, unattractive, and antisocial (19). In fact, there is a constant re-definition of disclosure rules that govern the social acceptability of disclosure in different contexts, with the rulemaking being a continuous process (20). A new set of such rules was brought on with the advent of social media and ubiquitous internet access, providing less friction to self-expression and an easier, broader reach in terms of social connection. Computer-mediated communication, and social media in particular, may be an outlet for self-disclosure of loneliness and formation of new connections and interactions that may help alleviate this epidemic (21). In the early days of mass adoption of social media, it was cautiously hailed as a way for young people to develop self-esteem and improve psychological wellbeing (22–24). However, it is still not clear whether social media has a net benefit or harm for mental health (25, 26).

There are various gender differences as well as similarities on how loneliness affects men and women. A meta analysis of nearly 400,000 individuals showed across the lifespan, mean levels of loneliness are similar for males and females (27). However, another large study of participants in 237 countries showed that loneliness increases with individualism, decreases with age and men are lonelier than women (28): “the most vulnerable to loneliness were younger men living in individualistic cultures” (p. 4). On the other hand, smaller studies have other findings. For instance, a study on gender differences of loneliness in adolescents in Jakarta suggested women are more social and emotionally lonely than men (29). Thus, it is not obvious which gender would experience more loneliness and express it on social media.

Measuring self-disclosure is further complicated by an uneven willingness to express feelings publicly. There are numerous studies that show women are more likely to report loneliness than men (30) or admit to being lonely (31). On the other hand, social media use during COVID was shown to increase (32), with possible increase in sharing of both media and own experiences. As COVID-19 can be treated as a natural experiment, we compare self-expressions of loneliness in a year before and a year during the pandemic in order to address the following research question:

**RQ1**: How does the volume of self-disclosures of loneliness by women and men change during COVID lockdowns?

Past research shows that lonely people often communicate negatively and elicit loneliness from others (7). However, given differences in gender roles of men and women, the two genders may sometimes experience loneliness in different contexts. Although according to the evolutionary theoretical framework, loneliness is viewed as a heritable trait, gender differences are attributed less to genetics and more to environmental factors such as social norms and cultural differences (33). Self-disclosures on social media provide an opportunity to discover the experiences of its users and the context of loneliness they may be feeling. Thus, we ask:

**RQ2**: What are distinguishing topics discussed by women, and not men in loneliness disclosures (and vice versa), before COVID?

As COVID provided a natural experiment by enforcing social isolation, it can be further probed to discover how the topics change among the genders during social isolation, as past literature shows men and women have different ways of coping with loneliness (34). Further, during COVID the change in work and parenting modalities have been shown to shift to the traditional childcare arrangements, putting more burden on women (35) and increasing the likelihood of burnout (36). On the other hand, COVID-related mortality rate for men was 1.6 times as high as the death rate for women, potentially adding more stress (37). Thus, we ask:

**RQ3**: What are distinguishing topics discussed by women, and not men in loneliness disclosures (and vice versa), during COVID?

Finally, past literature suggests that women give and receive more social support on social networking cites than men (38). This might be even more so in receiving support when lonely, because men are likely to be viewed more negatively than women when self-disclosing loneliness due to social stigmatization (31). As the lack of connection with others is a cause of loneliness, and a possible remedy is online interaction and social support provided there, we examine the rate and types of responses that self-disclosure of loneliness on Twitter receives by asking:

**RQ4**: What is the rate of response to these posts for each gender?

To answer these questions, we contribute a dataset of 1.6 million Twitter^1^ posts in the span of two years around COVID-19, and apply a combination of quantitative and qualitative methods to understand the extent of loneliness self-disclosures by users likely to be of one or another gender, and to find the contexts in which loneliness is experienced by each. The findings of this study have important implications for possible tailored interventions, both using communication technologies, and not.

## 2. Materials and methods

### 2.1. Data collection

We began by querying Twitter Streaming API (39) for keywords “loneliness” and “lonely” for the duration of March 15, 2019 to March 14, 2021, without any other criteria (not limiting the collection by language, location, or any other field). We decided not to use the keyword “alone” as it was used in too many contexts outside of loneliness. The API returns only publicly posted tweets, and not direct ones between users or those posted by restricted-access accounts. We then used several filtering techniques to discard tweets which were not self-disclosures. First, 1500 tweets were annotated by both authors, for whether the mentioning of loneliness was indeed self-disclosure, where loneliness is defined following Paloutzian et al. (40) as “the perceived discrepancy between one’s desired and actual level of social connection,” and self-disclosure as explicit or implied attribution of the feeling to the self. The annotator agreement was measured on 119 tweets annotated by each author separately, resulting in the label overlap of 89.6% or Cohen’s kappa κ=0.845. Meanwhile, we found several instances of song lyrics and other art which had the keywords, which we noted for filtering. We further excluded tweets with URLs, retweets (reposts of other users’ tweets) and tweets that were empty after excluding special characters. Overall, 809 tweets (53.9%) were labeled as loneliness self-disclosure. Finally, we built a machine learning classifier to filter the rest of the data. The classifier is a Support Vector Machine, and it achieves precision of 0.71 and recall of 0.82, evaluated using cross-validation. A version of this data was used in previous studies on the loneliness self-disclosures in general population (41) and the replies to it (42). Note that because they keywords are in English language, the retrieved posts are also mostly in this language, but we also constrained the tweets in our data to those labeled as being in English by Twitter (a label that is provided by the API).

To identify the possible gender of the users captured in this data, we consider the name field in the profiles, which the users are free to assign to themselves. Note that this field has few restrictions, and one may use letters, numbers, spaces, and special characters, and of course the name may not be that of the user. Still, gender inference from self-disclosed names have been used for a variety of research, including demographic, economic, and academic (see (43) for a summary). Following previous literature, we use the name listings from US Social Security and the National Records of Scotland, supplemented by user names from Google+ (44). Note that, due to the limitations of these sources, it is not possible to infer non-binary genders, which is a limitation of this study. Upon applying the listings to the names, we discard any users who did not unambiguously match with a gendered name. To assess the accuracy of this approach, we randomly sampled 100 users and labeled them for gender using information in their user name, handle (the account name beginning with @), and description field. The genders assigned by the annotator matched with that assigned by the algorithm in 93% of the cases, having accuracy of 0.92% for female and 0.94% for male accounts. Inter-annotator agreement between the two authors, estimated on 30 tweets, was 83.3% (all users received a gender label, no “unknown” label was used). Thus, throughout the study we will refer to the two groups of users as “male” and “female,” but these are inferred from the users’ self presented name, which may or may not reflect their “offline” gender. Even though the use of false representation is forbidden by the Twitter Misleading and deceptive identities policy (45), it is possible some accounts are made with a false persona, leading to our next filter. Finally, note that here we are able to capture a person’s *self-perceived gender*, not *biological sex*, and throughout the paper we use *male/female* interchangeably with *men/women*, with this caveat in mind.

Next, we attempted to remove spam or disingenuous users by considering their posting rate (46). As each tweet we gather contains information about the user at the time of the posting, we use this data to calculate their posting rate: number of tweets they have posted divided by the number of days the account has been active (using user “created at” and “user statuses count” fields). We remove users who have posted 16 times or more per day (that is, once per waking hour) on average in the account’s lifetime. We also remove those whose number of tweets decreased in the span of our dataset (who have removed posts, made them private, or had their posts removed by the platform). Using manual checks, we verified that the vast majority of such accounts were potentially spam (many soliciting interaction on other websites while claiming to be “lonely”).

In summary, we apply the following filters: keyword filter to collect the data, machine learning classifier and list of non-relevant keywords and phrases to identify self-disclosure, name dictionaries for detecting user gender, and posting rate filter for potential spam accounts. This results in a dataset of 1,600,371 posts from 1,240,961 users: 664,423 female and 576,538 male.

The data collection and analysis were performed using custom Python scripts, with the aid of the pandas data analysis library^2^ and the Gephi network visualization tool.^3^ The code for data cleaning and analysis is available at https://github.com/ymejova/LonelinessAndGender.

### 2.2. Volume of posting and replies

We begin by examining the volume of the posted material to answer RQ1. We separate the two genders, and for each day we compute the number of unique users in our dataset (counting users instead of posts alleviates the dominance of users who may post many times within a short period of time). In the duration of the data collection, due to technical difficulties data collection has gone down. Thus, for the 19 days for which the data collection went down for more than 1 h, we interpolate the rate using values from the surrounding days (using linear interpolation). We also examine summary statistics of posting rate by each group.

To assess the rate of engagement with these posts and answer RQ4, we also examine the reply, like, and retweet statistics of the posts. Since the original data was collected in a “streaming” fashion, as the posts were being posted, their metadata does not include the subsequent events like retweets and likes. Thus, on February 22, 2022 we re-collect a sample of tweets (sampled uniformly in time and across genders) using the statuses/lookup Twitter API call, which provides the latest metadata for a given tweet ID. Note that this way we were not able to obtain updated information on tweets that were deleted or made private since the time of their posting. This way, we obtain retweet, reply, and like metadata for 13,338 tweets for female and 11,736 tweets for male users. We compute summary statistics and compare the distributions of the responses to these tweets.

### 2.3. Content analysis

In order to analyze the content of the tweets, we make use of Natural Language Processing to extract terms of interest. We begin by removing special characters (including smileys) and URLs, lower-casing and tokenizing the text (breaking it into words). We then remove the stopwords (provided by the python nltk library). This then allows us to compute the frequencies of different words in the self-disclosures posted by male and female users. To do this, we compute odds ratios (ORs) that measure the likelihood of a word to be used by one, but not the other group, as follows:

(1) $${\mathrm{OR}}_{w}=\frac{\left(W_{\;f}(w)+\alpha )(W_{m}(\neg w)+\alpha \right)}{\left(W_{m}(w)+\alpha )(W_{\;f}(\neg w)+\alpha \right)}$$

where $W_{\;f}(w)$ is the frequency of the word w in tweets posted by users identified as female, $W_{m}(\neg w)$ is the frequency of all other words posted by users identified as male, similarly for the other variables. The parameter α=1 is an additive smoothing parameter. Ordered by OR, those with highest OR can be interpreted to be used more by female users than the male. Reverse comparison can be computed similarly.

To answer RQ2, we compute such ORs in using the tweets posted in the year before COVID. To answer RQ3, we compute similar ORs, but using the tweets in the duration of the first COVID lockdown, between March 15 and June 1, 2020. Once the ORs are computed, we consider the top 100 words with largest ORs and plot a co-occurrence network visualization wherein a node is a word, and an edge is weighted by the number of tweets in which the two words occur. We plot these networks using a force-directed algorithm ForceAtlas2 (47), which positions well-connected nodes in the center, and less-connected ones in the periphery of the network. Finally, for visualization we scale the size of the words proportional to their OR.

To better understand the context in which these words are used by each gender, we sample up to 50 tweets for each word (uniformly at random, for the gender being considered) and use thematic analysis to manually code them for themes. The sampled tweets were first categorized into major categories, and then further refined into themes. The iterative coding process resulted in three overarching categories: describing context in which loneliness occurred (while parenting, at work, etc.), expressing other feelings alongside loneliness (anxiety, depression, boredom, etc.), and loneliness coping mechanisms (such as asking for gaming partners or watching movies). A subtle distinction between the context and coping mechanism is the element of purpose: contexts are those in which people find themselves lonely, whereas coping mechanisms are contexts into which people put themselves in order to alleviate loneliness (though this distinction in purpose may be difficult to distinguish in short writing such as tweets). Each of these themes was then elaborated on with 3–5 major categories for which example tweets were selected for the paper.

## 3. Results

### 3.1. Self-disclosure rate

**Figure 1:**
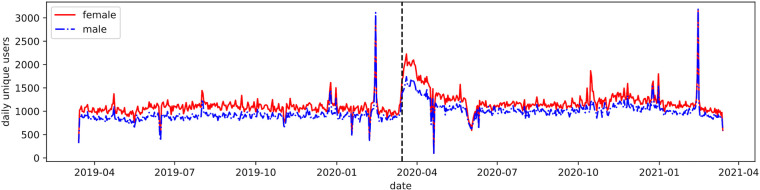
Daily number of unique users posting loneliness self-disclosures, by gender.

We begin by examining the rate of self-disclosure by the male and female users over time. Figure 1 shows the number of unique users posting loneliness self-disclosures over the two years; the start of the pandemic-related social distancing measures are marked with a grey vertical line. In reply to RQ1, we find a marked sustained elevation in posting by both genders immediately after March 15, 2020, when the first wave of lockdowns have happened in U.S. and internationally. The rate never normalizes to the pre-COVID average, and lifts again during the winter 2020–2021, when additional lockdowns were instituted in some areas. At the peak of the first lockdowns, the posting rate almost doubles, from an average of 1,062 (σ=172) in the week before March 15, 2020 to 1,950 (σ=194) in the week after for females and from 937 (σ=113) to 1,537 (σ=123) for males. The change in average posting is significant using one-sided t-test with p=0.

However, we note that there are more self-disclosures detected by female accounts than male, which is consistent with past studies reporting women more likely to admit being lonely than men (31). On average throughout the two years, 1,153 (σ=235) female users posted each day, compared to 978 (σ=204) male users. In terms of average number of posts per account, female accounts post slightly more tweets, at 1.29 (σ=1.01) than male at 1.26 (σ=0.98) (this difference is significant at p=0 using Mann-Whitney U test). This could be attributed to several factors: that female users simply feel more lonely than the male ones, but also that they may be more willing to share their emotions on Twitter, or that they are more likely to use their name identifiable as female (among several other factors that would be difficult to check without surveying the users themselves).

Considering the temporal peculiarities of the posting beyond the lockdowns, we find two peaks—both are during the Valentine’s day around February 14. Both genders have very similar posting dynamics over time: the Pearson correlation between the two posting volumes is ρ=0.94, suggesting that the two groups respond similarly to the world events at large, which is consistent with the finding of not significant differences between experiences of loneliness between genders across the lifespan (27).

Therefore to answer the first research question, although women are slightly more likely to self-disclose their loneliness than men, there is no significant difference between genders over the span of two years towards how they react toward societal changes, however female accounts do post more self-disclosures overall.

### 3.2. Content themes by gender before lockdowns

Next, we turn to the content of the posted self-disclosures. As both male and female subsets contain around half of a million tweets each, we use Natural Language Processing (NLP) to extract terms of interest. First, we must remark that the most frequently used words in these loneliness self-disclosures are quite the same for both genders: out of the 100 most frequent words, 92 in the period before, and 91 in the period during COVID are the same words between the two genders. These words include, of course, the query words *lonely* and *loneliness*, but also *alone*, references to feelings: *feel*, *feeling*, *sad*, and references to others: *friends*, *people*, *someone*, *love*, etc. We refer the reader to previous works on the major themes of loneliness self-disclosure in non-gender-disaggregated version of the data (41, 42).

**Figure 2:**
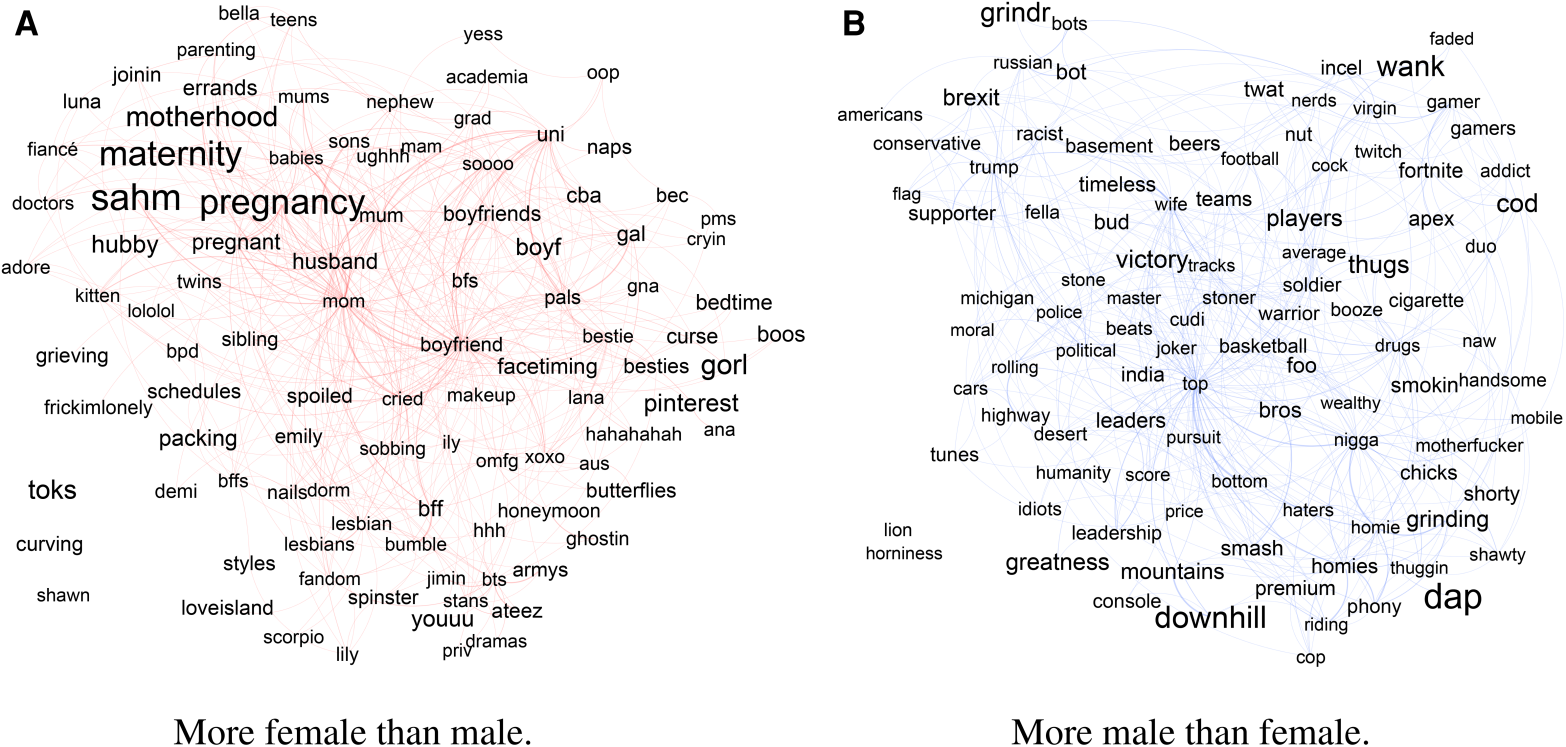
Top 100 words (left) used more by female than male, or (right) used more by male than female users, by Odds Ratio, in the year before COVID. Layout by force-directed ForceAtlas2 algorithm. Size of words is proportional to their Odds Ratio. Links represent co-occurrence in a tweet.

Instead, we turn to peculiarities in the way the two genders express their loneliness on Twitter, as captured using Odds Ratio (OR), which measures how much more likely a word is to be used by one, but not the other, group. Figure 2 shows the top 100 words (left) used more by female than male, or (right) used more by male than female users, by OR. Recall that the layout by force-directed algorithm, which puts the most connected words in the center. Size of words is proportional to their OR (the exact ORs are available in the Supplementary Material). Note that these were computed on data before COVID, and could be considered a baseline expression of loneliness in “ordinary” times. Below, we describe the major themes of the words more likely to be used by one, or the other, gender. We group discussion by the above-mentioned themes, but it is important to note that the categories are interconnected and through simultaneous coding a single tweet can fit several categories at the same time. The main themes are summarized in Table 1. Below we focus on the major themes present in the content and quote select posts. We leave the theoretical and policy implications of the findings to the Discussion section.

**Table 1:** Summary of themes.

Contexts	Emotions	Coping
Women		
Motherhood	Anxiety	Meeting apps (Bumble, Tinder)
Friendships	Borderline Personality Disorder	Social media (TikTok, Pinterest)
University/Academia	Premenstrual Syndrome	Music fandom
* Living alone	Anorexia	Therapy, support groups
* Remote learning/teaching	* Anxiety around annoying	Pets, plants
* Career progression	others by reaching out	* Facetime/Skype, messaging
* Death & illness in the family	* Burn out	* House party
* Being an essential worker	* Gratitude	* Chocolate
Men		
Gaming	Suicidal ideation	Meeting apps (Grindr)
Politics	Incel culture	Social media (Twitter, Instagram)
Leadership	Sexual frustration	Streaming/media (Twitch)
* Inquiries around rules & travel	Motivation/achievement	Drugs & alcohol
* Being away from friends & family	* Overwhelmed	Pornography & masturbation
* Societal origins of loneliness	* Porn addiction	* Watching TV
		* Playing musical instruments

Those preceded by * are more prominent during the COVID period.

#### 3.2.1. Loneliness-associated themes more likely from female users

As can be seen from Figure 2A, in loneliness self-disclosures by accounts identified as female, family is a prominent setting in which loneliness is often mentioned, including during pregnancy, as a stay at home mom (“sahm”), maternity leave and around parenting in general. Bond (48). The second largest context is close relationships, husbands, boyfriends and other relationships are also mentioned (best friends, as well as “lesbian”). This finding is consistent with the literature of the gender differences in self disclosures online, and women being more likely than men to self-disclose about their personal relationships and a variety of topics. The next context is around university, schooling, and academia. These are followed by loneliness during illness, travel, and holidays. In terms of psychological correlates, anxiety, depression, premenstrual syndrome (pms), and borderline personality disorder (bpd), as well as mentions of sleep deprivation, disability, and addiction. Finally, several coping mechanisms were mentioned: the posters often turned to social media (especially Bumble and Pinterest), support groups and therapy, pets, make up and nails, as well as fandom of various music groups. We discuss the each of the themes in more detail below.

##### 3.2.1.1. Contexts around loneliness

Pregnancy and motherhood figures prominently as a context of loneliness, partially confirming the efficacy of name-based gender inference and providing further support to evolutionary theory of loneliness and the gender difference coming from social roles (33). Overwhelmingly, the posters disclose their surprise at how *“no one talks about how lonely pregnancy is”* or how they *“Didn’t realise maternity leave would be so lonely and boring.”* There is also considerable lament over the loss of friends and usual social connections, while others reveal feeling lonely even when family and friends are around. Many find that loneliness as a parent is not discussed enough: *“Adulthood/ Motherhood can be so isolating and lonely and I dont think its talked about enough. So hard to make friends & keep them.”*

For stay at home moms, there are various feelings associated with loneliness, such as guilt: *“Only SAHM around my neighborhood. Extremely lonely, guilt ridden, questioning every decision.”* or loss of identity: *“Being a mom is so lonely. Especially as a SAHM. Its like you lose your whole identity.”* Some voice visions of alternative situations: *‘Being a sahm is lonely and depressing at times say what y’all want but sometimes I wish I had a job or wish I could go to college just so I could get out and socialize and (I don’t mean to sound like a bad mom) but get a break from my baby.”* Those on maternity leave also echo similar sentiment: *“Back to work in a month and I can’t wait to see people again. Apart from a handful of lovely people that have bothered with me since being on maternity leave, most days have been pretty lonely.”* Along with parenting, other difficulties are sometimes mentioned: *“Listen, I know parenting is HARD and lonely and exhausting. I get it. I’ve parented in grief, parented with depression, parented for weeks on my own, parented special needs and parented a lot of kids at once. It’s not easy.”* Although others express thankfulness for the support they received from their loved ones: *“Even tho being a sahm gets so lonely and depressing, I’m truly so grateful my man provides for us. Never go without needing or wanting. My hard working Bebe I can’t wait for him to get home.”*

The next category includes close relationships with romantic partners, friends, and siblings. Changes in close relationships and losing a connection with someone was a major category that women discussed. In relation to husbands for example: *“I’m lonely. Missing my hubby. I don’t talk to anyone at work, hardly get to talk to him, and my family is usually very busy. I feel so alone I haven’t had decent human interaction in days and it starts to hurt your soul.”* Similarly to being away or disconnected from romantic partners, women lamented being away from their best friends: *“WOW Im sad and lonely cos my bff has gone home and I’m stuck here for another week someone help me before I go insane w/o him to keep me grounded.”* Beyond these relationships, some users mentioned non-traditional relationships, such as polyamory: *“I’m either single and lonely or getting involved with multiple people roughly simultaneously and have to balance time and attention and try not to make anyone feel less important or loved. Poly curse? Poly curse.”* These findings of women self-disclosing loneliness on SNS in the context of relationships with others is consistent with previous research (48).

Another prominent context around loneliness is university and academia. Students at the university often lament the distance between them and their family, friends, and significant others: *“being away from home at uni is so lonely i hate it”*, even joking about dropping out: *“Might drop out of uni from loneliness hehe who knows :).”* Although many students may experience loneliness, their teachers and older peers also appear in our sample. Those in post-graduate programs discuss workload balancing: *“Post-grad life has me struggling to find the happy medium between not being bored/lonely and not having so much stuff to do that I wish I was bored/lonely”* and making career decisions: *“also just wanna talk about the scary whirlwind of trying to figure out what I’m going to “do”—post grad is a very weird, lonely, and deeply personal experience. It’s easy to graduate with a “sense of direction” but this new void opens as soon it’s over and man I’m just lost.”* Some women who are trying to make a career in academia share their feelings in the face of uncertainty: *“I have struggled with feelings of loneliness since moving to […], and after celebrating my dissertation proposal alone, I felt I didn’t belong here. I thought I was wasting my supervisor’s time by staying, particularly given that my future in academia is uncertain…”* and with few job prospects that require personal sacrifice: *“Lately, I’ve been thinking a lot about how I moved across the country for what was essentially my only chance at my dream job. And like, academia is very lonely if that wasn’t already clear […]”* Although there are positive stories as well: *“TA/grad student story time! My freshman fall quarter I took an intro biology class. I was nervous and lonely and unsure of myself. My TA […] was so passionate and encouraging that she bonded a whole class of freshman through evolution and ecology.”*

##### 3.2.1.2. Emotions around loneliness

Further, the sampled self-disclosures also included other emotions and even possible medical conditions. Anxiety, including social anxiety, is often mentioned as a companion of loneliness, *“I feel so so incredibly lonely when I push people away but having people close to me just involves so much anxiety and constant fear they will start to hate me and just leave. #bpd #bpdfam.”* Mentioned here, borderline personality disorder (bpd) is when a person may experience intense mood swings and feel uncertainty about how they see themselves, which can hurt family and work life, the ability to make long-term plans (49). As women are more likely than men to have it, it is not surprising to find it in the terms used more by accounts we identified as female than male. In fact, some posters directly address the difficulty they have in communicating with others: *“I only have one friend and you guys will have no idea how lonely that makes me. It’s even worse having BPD as I’m paranoid that my friend secretly hates me so sometimes I get tempted to never message them again! I don’t want to annoy them. I wish people would be more straight up.”* Yet others associate loneliness with premenstrual syndrome (pms): *“In the last week I felt extremely lonely. I have a feeling that pms caused it for me, but knowing this did not make it easier to handle.”* We also found mentions of anorexia, and not necessarily negative ones: *“anybody wanna message on kik or something? we could be ana buddies or we could just talk about anything. i’m severely lonely. […] #anabuddies #anacoach #proana #edtwt.”* Further, sleep deprivation was often mentioned, along with excessive social media use: *“sometimes i just watch tik toks bc everybody is asleep and i’m lonely.”*

##### 3.2.1.3. Loneliness coping strategies

While expressing these feelings and concerns, the posters mentioned possible ways to alleviate their loneliness. The app Bumble is often mentioned as a possible solution—its BFF mode allows users to find platonic friends. Among its mentions, we find some positive reviews: *“Can I just say how BADLY I’ve been struggling with anxiety recently and @bumble bff is actually a real nice release. So much female positivity!!! Cute girls wanting thinking I’m cool!!! I love it. If you’re ever feeling distant or lonely defo sign up to it”* and also negative (in this case, perhaps for romantic relationships): *“Before i redownload bumble and have dozens of meaningless conversations that don’t lead to meeting in person because i feel like the conversation is forced and not authentic but im still lonely so i try again every couple months, does anybody wanna admit they have a crush on me.”* Other apps, including TikTok, Tinder, and Pinterest are mentioned, however also that these often don’t help with loneliness: *“Pinterest makes me feel ugly fat broke lonely and uncreative all in one scroll I’m deleting the app.”* Another prominent possible remedy is fandom of music bands. BTS, also known as the Bangtan Boys, is a popular South Korean boy band whose fanbase (“army”) uses their common interest in the band to find connections and alleviate loneliness, for instance: *“okay texan armys going to the dallas show say yeehaw and let’s be friends bc ya girl lonely on this bird app #BTS_TEXAS #btsdallas.”* We also find more formal psychological help mentioned, including support groups and therapy. During Christmas, the users organized a hashtag #joinin where people spending Christmas Day alone can find company (50). Finally, many posters mentioned pets as important ways to deal with loneliness, with some considering them a part of their family.

#### 3.2.2. Loneliness-associated themes more likely from male users

In loneliness self-disclosures by accounts identified as male, we find loneliness to be mentioned in the contexts of video-gaming, politics, being a part of the LGBTQ+ community, and missing their wives (often after being separated by work, travel, or divorce). Alongside these, the tweets described associated psychological states: negative ones including self-criticism, suicidal ideation, addiction, and being bullied, and positive ones including motivation and competition. To cope with these feelings, the tweets mentioned seeking out like-minded people, connecting via social media (sometimes using premium accounts), interacting with bots and AI, and distractions including sports, drugs, and sex.

##### 3.2.2.1. Context around loneliness

As can be seen in Figure 2B, there are several clusters of keywords indicating particular circumstances in which loneliness is mentioned. First, gaming culture is heavily represented in the dataset, with words such as console and games including Call of Duty (“COD”), Fortnite, Super Smash Bros, and Apex Legends mentioned. The players of these games often seek companionship during the gameplay, especially “gamer girls”: *“Any gamer girls want to join :) we need more female players :) cause I’m lonely no girl friends only boys.”* Although team games present a potential social space, it is not clear that they alleviate loneliness, as many users explicitly state: *“[this game] makes life lonely.”* Still, there were signs that “gamers” are perceived as a community one may be a part of, for instance: *“Happy Valentine’s Day! We may be lonely gamers but we are each others valentine.”* Note that gaming may also be considered as a loneliness alleviation strategy, if individuals engage in it for this purpose. It would be necessary to employ additional analyses to gauge the directionality between gaming and loneliness, and the possible mediating factors.

Another prominent topic is politics, including mentions of then-American President Donald Trump, UK’s exit from EU (Brexit), and being “conservative”. We find many calls for like-minded individuals, and lamentations of being alone in one’s political stances, for instance: *“Just an ordinary Conservative member who feels lonely amongst the Hard Brexit types who dominate locally. Got a place for me?”* Some express a desire for a mate who would match their political views: *“And sadly I live in a largely democratic state, so I haven’t dated anyone in a long and lonely 14 years. But being lonely isn’t enough to get me to jump onto that crazy train. For now, I’ll continue to hope that eventually I’ll meet a nice conservative woman”* The political identities may also intersect with others: *“OK, so like, am I the only person who is a right wing conservative + LGBT? I ain’t here to start any arguments, I am just kinda lonely i guess.”*

Loneliness is also often mentioned around “leadership,” “greatness,” and “wealth,” associating success with loneliness. Some of these statements come from the gamer culture, with victors claiming that it is *“lonely at the top.”* Such statements are also contextualized in professional spheres, with some claiming loneliness is a part of it: *“Being in a leadership position, you can’t do what you used to do. You can’t leave early, you must stay late. It’s a lot of stipulations and it’s SO LONELY AT THE TOP. But I wouldn’t want it any other way”*; while others asking for more resources to help leaders: *“We expect our social sector CEOs to produce miracles w/ few resources. But it can be lonely & stressful at the top. Investing in support of nonprofit leaders is tremendously important.”* Yet others even literally lionized themselves: *“I rather be a lonely lion then a famous sheep.”*

##### 3.2.2.2. Emotions around loneliness

Besides describing the circumstances around one’s loneliness, the posts also express an array of accompanying emotions. Although we have seen loneliness being put in a positive light in the context of leadership and achievement, many more emotions disclosed in these posts were negative, some even mentioning suicide.

Among such posts, many included self-criticism: *“Sometimes I wonder why I’m lonely and then I look in the mirror and I remember that I look like this. […]”*, as well as suicidal ideation: *“Im lonely and I want this shut off. If I kill my self Ill kill two birds with one stone.”* Some attribute it directly to loneliness: *“Loneliness is EVERYTHING it’s cracked up to be. What few people I’ve met since either wants just a one night stand, financial support or booze and drugs…Why am I still here? No one knows I exist.. been suicidal for months now…”* Yet others would refer to themselves, perhaps in a joking or hyperbolic fashion, as “incels”—a term used for “involuntary celibate” men, often associated with a misogynistic stance (51). What is the relationship between loneliness and the incel subculture is an interesting research question. In fact, “virgin” is one of the keywords particular to the male users, often used in a lamentation such as “Lmao Im so lonely, such a virgin,” alongside with others having to do with sexual context (“wank,” “nut,” “cock”).

##### 3.2.2.3. Loneliness coping strategies

The self-disclosures also come with potential coping strategies suggested by the posters, mainly to engage virtually on social media and gaming platforms, but also some concerning ones around drug and alcohol use.

Grindr, a dating app for LGBTQ+ people, figures prominently in the words more likely to be used by male Twitter accounts, suggesting it is used less by the accounts we identified as female. It is often cited as a resource for finding companionship, though not always effective: *“tbh being gay in a small ass hick town can be lonely as fuck like the dating pool is my ex and a handful of 50 years old men on grindr. nothing to work with smh.”* Though some users question whether the app is the solution to loneliness: *“I think the reason I give in and redownload Grindr is simply because I get lonely and foolishly think that using that app will fix that feeling.”* Yet other self-disclosures speak of their decision to speak out about their sexuality: *“i Really Want to Be Out but. i want college to be as easy a process as possible, so staying in the closet would help. BUT i’ve had 2-0 friends for the last uhhh 5 years—friends as in accepted i was trans and were Actual friends. and i’m LONELY AS HELL. WOULD LIKE TO HAV BROS.”* For additional analysis of LGBTQ+ loneliness disclosure on Twitter, see Mejova and Hommadova Lu (52).

The posts also mention Twitter, Instagram, and Twitch, a popular interactive livestreaming service. Interestingly, people even mentioned interacting with bots on these platforms, out of loneliness: *“im so lonely one of these days im going to message back one of those russian sex bots that dm you on instagram.”* Opinion on AI-enabled bots is split, some mentioned the inclusion of AI bots in games making them lonely: *“Whats worse is that some game can now add AI to dungeon teams like a mercenary or something like that…makes me even more lonely”*, yet others wonder if AI could help solo gamers: *“Well I know it’s not cod [Call of Duty] I’m just saying it’s far too lonely without anyone else to play with. If we had ai they could help.”* Outside of social media and gaming, the posts mentioned other activities, such as watching sports, drinking with friends, and listening to music as distractions or outlets for their loneliness.

Another concerning connection that is often mentioned in these self-disclosures is between loneliness and drug use and addiction. Alcohol is often mentioned as an escape: *“I fucking LOVE booze. It numbs loneliness ;) ”*, as well as smoking: *“on my days off I just be smokin and jammin by my lonely.”* Others recovering from drug addiction say that loneliness can be a trigger: *“As an addict in recovery my trigger is loneliness. A good woman is hard to find. I finally noticed a trigger. Being alone. After weeks of realizing my fate, dope will at least take my mind of it. Terrible really.”* Yet others consider it a price worth sobriety: *“i stopped drinking/ smokin/ doin drugs & i literally lost every person i thought was my friend. & it’s lonely but i swear i wouldn’t have it any other way. fuck anyone who wasn’t really there for me.”*

Finally, a distinguishing theme in male account posts is the references to pornography, sex, masturbation and being a “virgin” (as mentioned in emotion theme above). For some, it provokes the feelings of loneliness: *“I’m so fucking single, I was watching porn and I had to stop the video because they kissed and I felt so lonely. I then remembered why I was, in fact, watching porn.”* Some disclose the need for masturbation only due to their lack of connections to others such as: *“I only jack off when im sad and lonely, as soon as i make a friend or get any kind of affection i dont feel any need to have a wank at all.”* While others admit a reliance on pornography built upon constant loneliness: *“I feel like even if I got a gf, nothing would change, because I’ve built this massive reliability on porn because of constant loneliness.”* For others, loneliness prevents them from enjoying sex: *“tfw you’re so lonely the nut don’t even feel good.”* We note that in the female sample there is 1 tweet mentioning porn, and it may be a gender mis-classification, emphasizing the one-sidedness of this topic. Instead, they may speak about sex in different terms, but none so distinct that they appeared in our selection of top 100 words.

As a final note, we mention that while examining these posts, we find many uses of online slang and grammatical peculiarities, which may limit the usefulness of NLP technologies trained on text written in a different style. Examples of such conversational language are calls for people to “dap” someone up (fist bump), references to women as “shorty,” and various gamer slang.

### 3.3. Content themes by gender during lockdowns

Next, we turn to the self-disclosures of loneliness that were posted during COVID. In particular, we examine the posts from March 15 to June 1, 2020, when worldwide lockdowns were happening. First, we will mention that the loneliness self-disclosures during this period of both genders gained new keywords related to COVID-19, including *quarantine* and *lockdown*. Again, we refer to previous work on gender-agnostic analysis of loneliness self-disclosures (41, 42).

**Figure 3:**
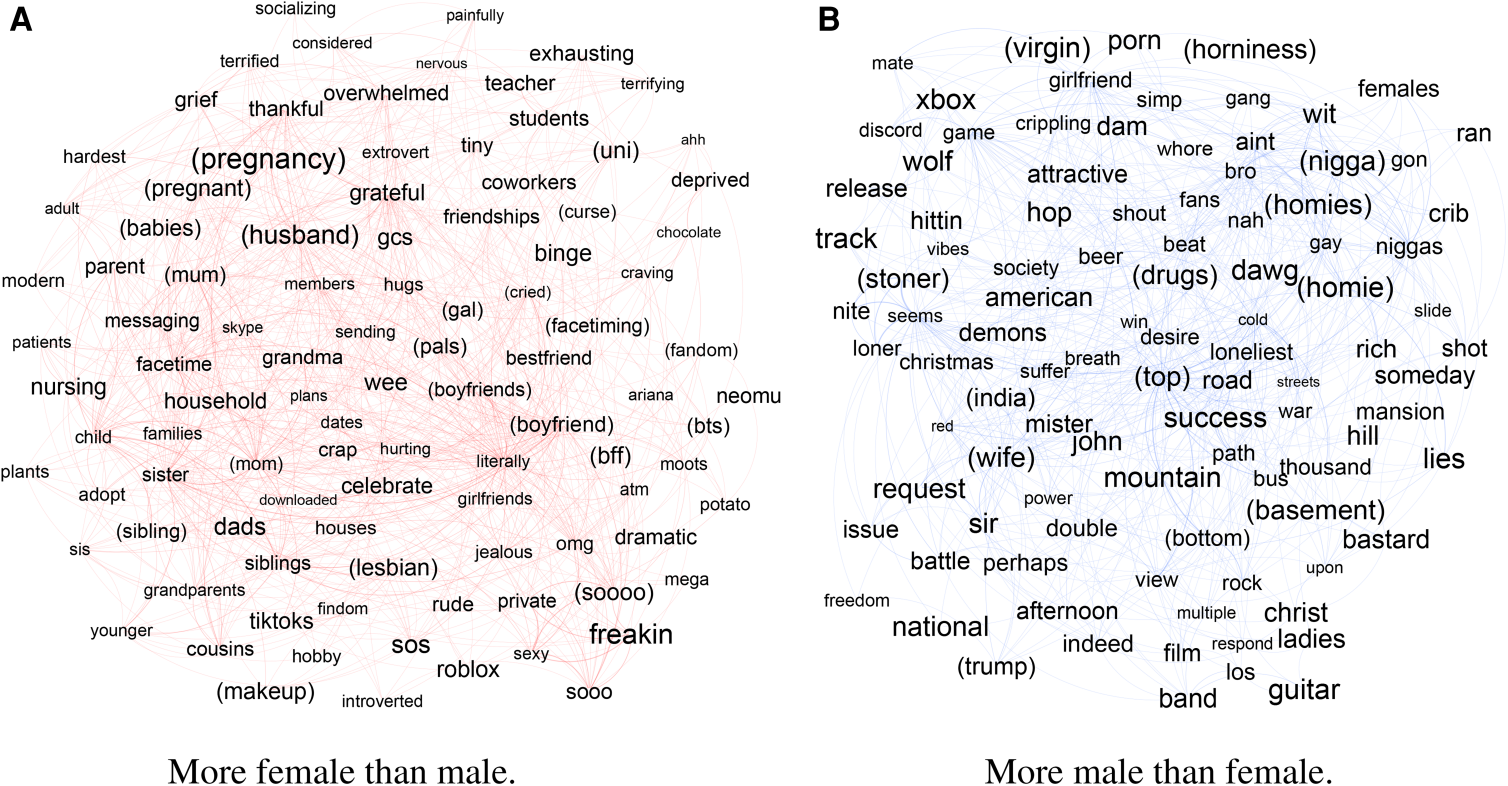
Top 100 words (left) used more by female than male, or (right) used more by male than female users, by Odds Ratio, during the first COVID lockdown. Layout by force-directed ForceAtlas2 algorithm. Size of words is proportional to their Odds Ratio. Links represent co-occurrence in a tweet. Words in parenthesis have also occurred in the top 100 words in the pre-COVID period.

In particular, here we examine the gender-specific themes associated with loneliness during the first 2.5 months of the pandemic-related restrictions. Figure 3 presents the top words mentioned (a) by female rather than male and (b) by male rather than female accounts, displayed similarly to Figure 2 (again, the exact ORs can be found in Supplementary Material). This time, the words that also appeared in the top 100 for that gender in the preceding year (in Figure 2) are shown in parenthesis, so that all words that do not have parentheses are new.

#### 3.3.1. Loneliness-associated themes more likely from female users during lockdowns

The accounts we identified as female continue to speak about family, pregnancy and motherhood, and their relationships. The coping strategies for loneliness—including that during quarantine—are also similar, including adopting pets and buying plants, using dating apps and gaming (as well as teaching family members how to play games), and more solitary hobbies and binging TV shows. Physical and virtual spaces become more outlined, including online messaging and Facetime (which are used for socializing, including dating), but also those which can be used for socialization within the constraints of quarantine, such as backyard parties.

##### 3.3.1.1. Context around loneliness

The contexts mentioned in these self-disclosures, do change dramatically. Although pregnancy and family life remain popular, many more are lamenting living alone, and express envy at those who have company during lockdowns; those with children at home also express a yearning for more adult interaction; and university students look forward to going back to the university campuses. For instance: *“A sad lonely bitch vent: I’m jealous of everyone home with their husbands during quarantine doing all these cute home projects and getting in family time. I miss my husband so much.”* For pregnant people, the distancing measures further exacerbated their isolation: *“my mom has big COVID 19 symptoms and they won’t test her because she’s “low risk” so she just has to quarantine and I can’t be around her due to being pregnant and high risk and that makes me so sad. She texts me abt how lonely she is. I’m gonna go cry.”* The teachers, on the other hand, express frustration about the lack of interaction afforded by the online teaching environment: *“Zoom office hours (with no students logging in) is lonely.”* Some lamented that the pandemic affected their career progression: *“I would’ve started my masters degree and now I’ll probably have to postpone until next year because all my exams have been cancelled and the uni I wanted to play changed their due date by 10 days only. Also I’m lonely and I feel worse.”* Even worse, some of those posting have experienced illness and death in the family, or are themselves in a hospital: *“I’m in a non-Covid hospital ward and none of the patients can see relatives, yet they all need urgent care—it’s a sad and lonely place. #covidfirsts.”* Those who continue to work, especially as “essential” workers, also express their isolation: *“Being an essential worker is lonely and miserable and scary. I no longer feel guilty for not just being grateful to have a job. This sucks.”* Yet some are trying to make the best of the time, for instance activists were using Twitter to celebrate Pride month despite the quarantines: *“Let’s turn all this lonely and boring quarantine to a gay and joyous one as we celebrate Pride Month 2020 virtually! It’s the time of the year again to raise our voice and this time, we’re taking it on another level! #EMPRESSive2020 #PrideMonth2020.”*

##### 3.3.1.2. Emotions around loneliness

These contexts were accompanied by a variety of emotions we have already encountered in pre-COVID posts, along with a few new themes. As previously, loneliness is often associated with anxiety attacks and depression. During lockdowns, the anxiety also included the fears of reaching out to others and annoying them: *“quarantine rly got me feeling lonely and terrified of annoying my same 7 friends all the time but thats what i get for not keeping up my other friendships.”* Those in already challenging situations start mentioning being overwhelmed with the increased isolation: *“been in isolation with a sick 6 year old for a week now. Trying very hard to not let my depression and anxiety overwhelm me. I thought being a single parent was lonely before but this is overwhelming.”* Others who continue to work also report burn out: “stress with work, feeling burnt out with so much on my plate, mixed with loneliness from this quarantine…. trying my hardest to not slip into a d*pr*ssive episode but damn.” However, alongside loneliness self-disclosures we find a theme of gratitude. Some are grateful to have a safe home to stay, others for their close ones and even pets who keep them company, and yet others for the internet communication that keeps them connected: *“Finishing this super productive day with a family FaceTime. Dinner and dishes were less lonely tonight. Thanks to facetime! Keeping us connected even when we’re apart. Perfect end to a long and busy day, so grateful for my loved ones and being able to connect with them online”*

##### 3.3.1.3. Loneliness coping strategies

One of the most prominent coping strategy we found mentioned by female accounts was using FaceTime for maintaining connection with family and friends. Other modes of communication have also increased, such as text messaging: *“im sorry if I started messaging you too much I haven’t left the house in over 30 days im so lonely this isn’t even a meme it’s just R E A L.”* Another popular strategy is binge watching TV shows, sometimes with the sole purpose of escaping reality: *“I was never one to binge shows but I can’t go anywhere and I’m getting so lonely so I just sit here in front of the tv for hours to avoid thinking about literally everything.”* Perhaps relatedly, many mention chocolate as a comfort food (also related to romantic love): *“Contemplating just really leaning into the loneliness today and marathoning romance movies and eating chocolate.”*

#### 3.3.2. Loneliness-associated themes more likely from male users during lockdowns

Figure 3B shows the terms more likely to be used by male users than female. Similar to the case of female accounts, the general themes remain the same: motivational mentions of success, gaming, references to others (*“nigga”*, *“homies”*) and some political banter. However, we see a diminishment in words associated with drugs and politics, and increase of those associated with seeking friendships and girlfriends. Moreover, unlike the female keywords, those posted by male accounts speculate on societal causes of loneliness and systemic issues.

##### 3.3.2.1. Context around loneliness

Besides the contexts we have observed before COVID-19 that included request for gaming partners and general expressions of loneliness, several new contexts have been mentioned in the period of lockdowns. Some have experienced job losses or slowing due to the pandemic. Interestingly, we found many people addressing politicians asking whether it is possible to travel, how long the lockdowns would be etc.: *“Respected Mr Modi I kindly request you to please don’t forward up to Curfew date 14 April’s, because I am feeling so lonely in home”*—it is not clear whether such requests reached the policy-makers or whether any replies were forthcoming. Some were away from family in other country: *“@PMOIndia @HCI_London @MEAIndia @meaMADAD struggling here and desperate to go back to India, missing family badly and feeling very lonely, I have lost my Job over shutdown in UK difficult to survive, its a mental torture, please get the flights started, ready to quarantine in india.”* Yet other posters lamented the closures of venues and events, such as those in music bands: *“It took away my only love; Performing. Cost me a bundle in cancelled shows. I’m lonely as hell. Depressed. Haven’t played guitar in a month.”*.

Interestingly, not only did the posts discuss the personal effects of loneliness, some expanded on the role of society in creating conditions for loneliness. One, for instance, pointed to capitalism: *“I know that it’s almost impossible not to feel isolated in a society that pits people against each other for profit. My work is to dismantle my loneliness by dismantling the systems that thrive from it. Feelings of lack and worthlessness are an affront to my natural way of being.”* Another argued that loneliness is imposed by society: *“Sometimes we do feel lonely but what if a lot of the times we’re sad while alone because society (movies, books, etc) has conditioned us to think we need to always be surrounded by friends? As an autistic person, I often feel conflicted by this. I wish to be alone but not sad.”* Yet another would like to find their role in the society: *“Social participation is an important need. Everyone has to find a role in society and makes one’s life meaningful. In this lonely city, I have to find my role. #HomeQuarantine.”* One observed that there simply isn’t a system to deal with loneliness: *“Broadly, we have systems to treat war wounds. And we have systems to treat hunger. But we don’t have systems in any meaningful sense to treat loneliness. And we’re now inflicting it widely. It’s the right thing to do. But it’s painful and will only get moreso.”*

##### 3.3.2.2. Emotions around loneliness

Similar to the pre-COVID period, the mentions of loneliness coincided with other emotional experiences, such as those around anxiety and depression, feelings of inadequacy, vulnerability, and exhaustion. Self-criticism abounds in such mentions, including around attractiveness: *“I wish I was attractive enough for an attractive woman to want to touch me or be close to me. So tired of being lonely.”* or being a “loser”: *“I’m so lonely and every time I try looking on dating apps I feel like I’m not good enough for anyone. I’m struggling to get my life back on track and no woman will ever want a loser like that.”* A combination of emotional difficulties make some feel overwhelmed: *“I wanna say this is true for me but I’ve been feeling weaker than I have in a while. Depression is gripping onto my future. The loneliness isn’t a battle for me anymore. Motivation has been an issue in schoolwork, once I start feeling overwhelmed by an assignment I shut down.”* Yet others mention porn addiction, even if half-jokingly: *“I may not show it But this porn addiction do be depriving me of important nutrients and testosterone while fuelling my loneliness.”* Still, not all emotional content is negative, as in the previous time segment, there are motivational posts as well, such as: *“Success can be lonely that’s why a bus has 50 seats but a rolls Royce only has 4”* (although such posts are not explicit disclosures of loneliness, and could be considered less relevant to this study).

##### 3.3.2.3. Loneliness coping strategies

The coping strategies suggested by the male accounts to lockdown-induced loneliness are very similar to those posted by the female ones, including watching TV shows and movies, talking to strangers, and making connections on dating apps (however explicit mentions of talking to friends and family on real-time video chat applications are not nearly as popular as for the female accounts). For some, the quarantine loneliness prompts them to get back with their previous partners (“exes”), but others advise against this. Some lament that the online friends have not resulted in real friendships: *“It’s deep. 50k/100k followers but still lonely as f. If the internet gets shout down today. People would realise they have no real friends because all that was seen was a race for perfection. Make real friends not just followers.”* Some mention picking up their instruments (often a guitar) and looking to start a band. One explicitly mentions music education as a solution to boredom: *“If only the schools still taught art and music. Every one of these lonely jack-offs could have learned to play an instrument and formed a band we could all never have to listen to. They would have been thrilled to be locked up in their garages together and not have to go to work.”*

### 3.4. Response

**Figure 4:**
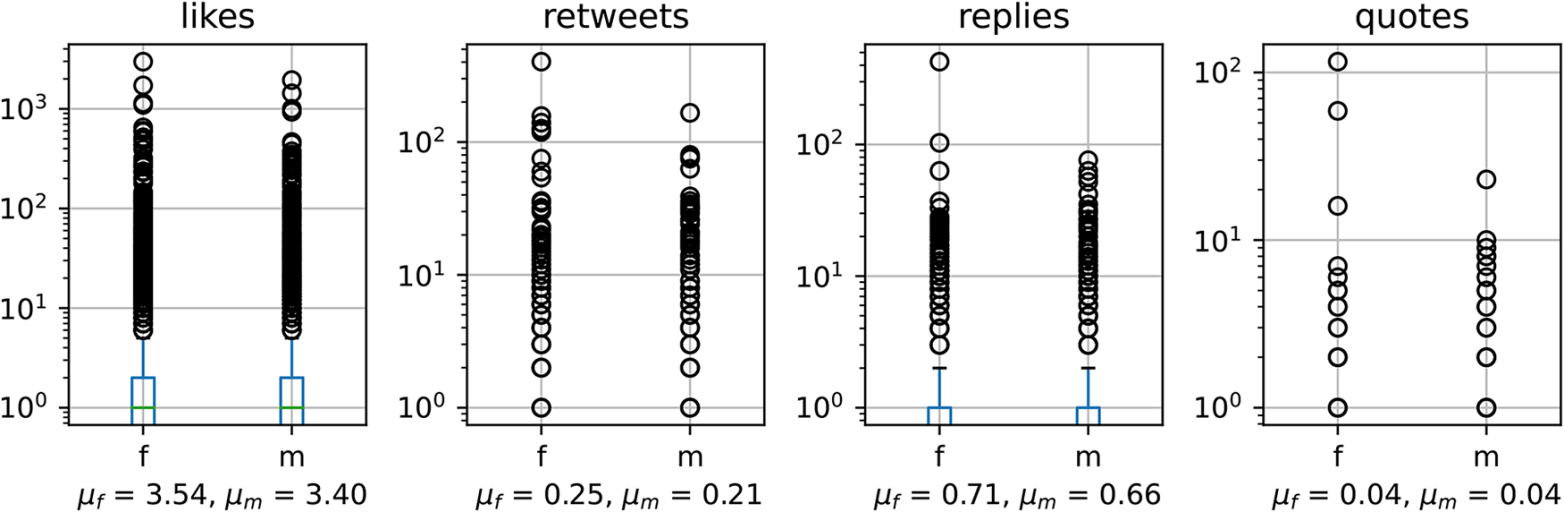
Statistics of four kinds of response to loneliness self-disclosures, by gender. Mean is specified under each plot. Note y-axis is in log scale.

Finally, we consider the various ways in which users may respond to the self-disclosures of loneliness on Twitter, which may be by “liking” a tweet, retweeting it (re-posting without additional content), replying to it (with additional content, directed to sender), or quoting it (with additional content, not directed to sender). Figure 4 shows the distribution of these actions for tweets by female and male users. Overall, we find a long-tailed distribution for all of these actions, as is often the case for social and real-world phenomena (53). Median for all statistics is 0, except for likes, which reaches a median of 1. These figures suggest that self-disclosures of loneliness on Twitter are, on average, unlikely to receive response. As can be seen from the figures, female users tend to have outliers with more responses than the male ones. Indeed, when we compare the distributions of these statistics using the non-parametric Mann-Whitney test, the likes are statistically different at p<0.01, and retweets and replies at p<0.05 (quotes are not statistically distinguishable). Thus, female users may receive slightly higher number of responses, but this may be dominated by a few outliers. It is important to note that overall over half of loneliness disclosures are had zero replies, leaving the majority of both men and women who disclosed their loneliness with no consequent social support.

## 4. Discussion

The findings in this study have important public health implications in the way loneliness is experienced by males and females, pointing to possible promising avenues of intervention. Further, many can be interpreted through the evolutionary lens, in terms of the purpose of loneliness to motivate action and reinforce social connections in order to propagate the species. We discuss each major finding in turn.

More female Twitter accounts self-disclosed loneliness, both during and before COVID-19 pandemic, however thousands of male accounts also posted their experiences with loneliness. Surveys have shown that women have reported increased levels of mental distress and loneliness during COVID-19 than males (54, 55) (although some studies find that race and income may also play a role (56, 57)). As some of the most successful interventions to alleviate loneliness have been shown to be cognitive behavioral therapy (CBT) (7), self-disclosure of the feeling may help in beginning the process of self-examination and re-evaluation of one’s thinking. Although our reply analysis puts in doubt whether social media is an interactive enough environment for an in-depth self-reflection to take place.

One striking result in the above analysis is that women in our data self-disclose loneliness throughout the parenting journey—from pregnancy, to baby rearing, to maternity leave or being a stay-at-home-mom—even before the pandemic. Literature suggests that during the pandemic, the responsibilities of child rearing and home-making were still largely laid on women’s shoulders, erasing years of progress to gender parity in the labor force (58, 59). Not only does having a child affect women’s careers, child rearing and working has been shown to be a “double-burden” in terms of health risks due to increased stress (60). This burden may be then passed along to the child, as mothers’ high loneliness was found to predict adolescent’s lower social competence, hostility and fear of negative evaluation (61, 62). Evolutionarily speaking, the increased attention of women to child rearing is a natural point of focus to ensure the survival of the child, especially when the child is small or the woman is in a vulnerable position of pregnancy. Although resources are available for mental health of new mothers (such as for Perinatal mood and anxiety disorders (PMADs), otherwise known as “postpartum depression”), or those with unplanned pregnancies, additional support may be necessary in terms of social interaction for pregnant people and parents of small children, even outside of the topic of parenting, in order to maintain a connection to society outside of the family circle. As numerous women on maternity leave disclosed being lonely, companies need to consider providing additional resources to women on maternity leave to decrease the level of social disconnect they feel.

Another common context of loneliness disclosed by female Twitter users is school: both being a student or a teacher or academician. Although women are more likely to finish their university degrees than men (63), females are likely to drop out of STEM fields due to hostile environment and lack of role models (64). Further, in academia, gender equity persists despite diversity initiatives (65). Counseling available on campus can help college students to cope with loneliness, and due to COVID-19 pandemic online counseling has become more common and accessible. Getting involved on campus might be another way to help deter loneliness, as research shows that students involved in extracurricular activities and less likely to be lonely (66).

Turning to the male accounts, a strong common context that we found around their posts was bonding over video games, seeking partners and teammates, discussing game releases and creating social circles on platforms like Twitch. Although about as many women are estimated to play video games as men (67), our data shows that men use it as a social activity more than women (although women do mention more games during the lockdowns). Competition and cooperation present in computer gaming is echoed in the evolutionary theory wherein individuals seek support in order to achieve goals and maintain a community. In the light of decreased necessity for pre-historic cooperative hunting or other more modern work activities, gaming could be considered as a cooperative bonding experience. However, current literature suggests only a weak positive relationship between video game participation and loneliness (68), although anecdotal evidence does point to the possibility of maintaining social connections through games such as Animal Crossing (69). It is possible that gaming could be a stepping stone to other more effective interventions that build social relationships outside the game realm.

Another peculiar feature of the loneliness self-disclosure by male accounts was the use of loneliness as a necessary cost for success (often in professional life). This was then accompanied with mentions to materialistic wealth including money and “mansions”. Such encouragement to perceive loneliness as a part of success may lead to what scholars call “toxic masculinity” (70). Already, campaigns exist to help men receive mental health assistance, such as the Campaign Against Living Miserably (CALM)^4^ in Britain, where 75% of all suicides are male. However, such services may not address the underlying cultural factors that lead to the glorification of loneliness. It may be the case that some interventions must be conducted at a societal (instead of personal) scale, that would advocate for not presenting loneliness as a necessary sacrifice to the path of success.

Another trend in our data is the mentioning of drug use by male accounts. As the United States (and other countries) goes through an opioid overdose epidemic, if loneliness is a trigger for even a fraction of users, addressing it could lead to saving lives. In 2021, 71% of preventable opioid overdose death victims in the US were male, according to the (71), however, “since 1999, female opioid overdose deaths have increased at a faster pace than male deaths—1,608% for females vs 1,076% for males.” As loneliness may play a role in the “use of non-prescribed opioids,” interventions such as group therapy may help those who are particularly triggered by it (72). Thus, addressing loneliness may be a possible element of intervention in this vulnerable population, among those focusing on physical effects of addiction.

As the scale of the loneliness epidemic becomes evident, all possible interventions should be deployed to tackle it. Communications technologies could play an important role, both in supporting communities and promoting inter-personal communication, and as a connection to public health services via teletherapy (18). Interestingly, both genders (but especially men) mentioned AI as potential conversational partner, usually as bots (sometimes even those serving spam). Despite sometimes lighthearted mentions of conversing with bots out of desperation, conversational agents could be increasingly possible with the fast advancement of AI and natural language processing (NLP) technologies. However, many challenges must first be overcome, including improved factuality, appropriateness of the advice, as well as a slew of concerns around confidentiality, privacy, and data safety (73). Still, the very existence of the dataset analyzed in this study is a testament that some individuals are comfortable disclosing loneliness on the Internet; whether they would also be interested in automated response is a fascinating research direction.

### 4.1. Limitations

This study has a number of limitations. First, the conclusions herein are limited to the platform (with its unique affordances and user base), the timing (2020 was a unique year), and the methodological choices of data selection and processing. The choice of keywords for data collection has surely excluded loneliness self-disclosures worded in a different way. On the other hand, the filtering techniques we employed to select self-disclosures were not perfectly accurate, and surely have mislabeled some tweets (both with false positives and false negatives). Although the authors have made the effort to provide hand-annotated training data for the algorithm, and hand-labeled gender classification output. Further, the limited human resources allowed the authors to examine only a sample of the posts. However, we used statistical metrics to make sure to select posts containing words most peculiar to each gender. Also, using available tools, we were not able to identify non-binary genders in our data, for which a more nuanced algorithm should be used (that likely will need more than a user name). Finally, as it is an observational study, we cannot claim causality (except perhaps the relationship between the beginning of lockdowns and the rise in loneliness self-disclosures). For instance, we cannot say whether the larger volume by female accounts is due to them feeling more lonely, being more comfortable expressing their feelings, or their use of recognizable names in their account profile. Complementary methodology must be used to tease out these relationships.

Finally, in this study we did not constrain data by location, as Twitter does not provide standardized location information (or it is almost always blank), and such a constraint would diminish the scale of the data. Nonetheless, we matched the Location field of user profiles to the GeoNames geolocation database,^5^ and found that we were able to match 50% of the data (note that it is a free-form text field, the user can write anything there). The largest match was with locations in the US (27.2%), followed by Great Britain (5.3%) and Canada (1.5%). The rest of the data has been geolocated to 237 countries. Given that the matches are not always accurate, some of these may be noise. Thus, it is likely that a large portion of the data is posted by users from the United States (especially if the location distribution of the unmatched users is similar to that of matched ones). However, a non-trivial minority of posts may come from other countries, which could have had a different lockdown schedule, resulting in some impact on the results.

Beyond the methodological shortcomings above, the nature of social media prevents us from considering a variety of additional omitted variables, including age, sexual orientation, individual character and values, socioeconomic indicators, and even familial status. Although tools have been proposed to infer some of these characteristics from Twitter profiles (such as age (74) and socioeconomic status (75)), their accuracy and applicability are not sufficient for large-scale analysis we aim for in this study. Older age, for instance, has been shown to correlate with self-rated loneliness across 25 European countries (76), although an even broader survey of 237 countries found that younger people reported more loneliness than the middle-aged (28). Moreover, relationship status was found to have significant indirect effects on life satisfaction though romantic, family, and social loneliness in Poland (77). Further, residents in socioeconomically deprived neighbourhoods were shown to be more likely to be lonely than the general population in Denmark (78). Cultural differences may also play a role, as the aforementioned global study found that people in individualistic (vs. collectivist) countries reported more loneliness (28). However, sexual orientation has been studied in the context of loneliness self-disclosure (52). In this study, we do not use this variable as control, as detecting social orientation on social media is a difficult task in terms of recall, and would result in a small subsample of the overall data, adding complexity to the methodological setup.

## Data Availability

Due to the sensitivity of the data (some of which deals with mental health and suicide), we do not make the posts available to download on the internet. This is also against the Terms of Service of the platform. According to these Terms of Service, we make the tweet IDs, the IDs of the posting users, and the gender classification available in Supplementary Material, which could be re-collected at a later date. Researchers interested in the complete data should contact the first author of the paper.

## Footnotes

^1^The platform is currently rebranding to “X,” but in consistency with previous literature, we use “Twitter” in this manuscript.

^2^ https://pandas.pydata.org/

^3^ https://gephi.org/

^4^ https://www.thecalmzone.net/what-we-stand-for

^5^ https://www.geonames.org/

## Supplementary material

Supplementary Table Odds Ratios of 100 words for each of the loneliness self-disclosure comparisons (the four word networks).
